# Editorial: Flavonoids and chronic metabolic diseases

**DOI:** 10.3389/fnut.2024.1403863

**Published:** 2024-04-22

**Authors:** Qian Zhou, Yueqiong Ni, Pan Liao, Yueliang Zhao

**Affiliations:** ^1^Shenzhen Key Laboratory of Food Nutrition and Health, Institute for Advanced Study, Shenzhen University, Shenzhen, China; ^2^Department of Microbiome Dynamics, Leibniz Institute for Natural Product Research and Infection Biology – Hans Knöll Institute, Jena, Germany; ^3^Cluster of Excellence Balance of the Microverse, Friedrich Schiller University Jena, Jena, Germany; ^4^Department of Biology, Hong Kong Baptist University, Kowloon, Hong Kong SAR, China; ^5^State Key Laboratory of Environmental and Biological Analysis, Hong Kong Baptist University, Kowloon, Hong Kong SAR, China; ^6^State Key Laboratory of Agrobiotechnology, School of Life Sciences, The Chinese University of Hong Kong, Kowloon, Hong Kong SAR, China; ^7^School of Public Health, Shanghai Jiao Tong University School of Medicine, Shanghai, China

**Keywords:** flavonoids, chronic metabolic diseases, gut microbiota, structure-activity relationship, omics

The escalating global prevalence of chronic metabolic diseases poses one of the most significant health challenges worldwide. These diseases, including obesity, diabetes, cardiovascular diseases, and liver disorders, can exist independently or interact with each other. This interaction significantly reduces the quality of life and leads to a heightened risk of mortality. Over the past few decades, continuous efforts have been devoted to the prevention and treatment of chronic metabolic diseases. Among various strategies, flavonoids have emerged as a promising candidate. Ubiquitously present in plants and characterized by their common C_6_-C_3_-C_6_ structure, flavonoids are recognized as beneficial components in human diets. They can be further categorized into subtypes such as flavonols, flavones, isoflavones, flavanones, and anthocyanins. However, further research is required to bridge the existing gaps in our knowledge. For instance, recent data indicate that the health effects of flavonoids may not be directly attributable to their molecular form, but rather mediated through changes in the composition, function, and metabolites of gut microbiota. Moreover, the application of omics approaches, such as metabolomics, genomics, and proteomics, holds tremendous potential for a deeper and more comprehensive understanding in this field, as compared to traditional methods. This Research Topic aims to enhance our knowledge of the diverse functions of flavonoids in the prevention and management of various chronic metabolic diseases. It particularly focuses on the application of emerging technologies and tools, such as omics, to reveal the underlying mechanism by which flavonoids exert their beneficial effects.

This Research Topic includes a total of five manuscripts, consisting of three research articles and two reviews. Yi et al. summarize recent advances in the use of flavonoids to improve type 2 diabetes mellitus and its complications. Flavonoids have been shown to regulate diabetic vascular disease, improve diabetic cardiomyopathy, ameliorate diabetic nephropathy, and mediate diabetic retinopathy, among other effects. The underlying mechanisms include decreasing insulin resistance, reducing oxidative stress, regulating glycolipid metabolism, and controlling gluconeogenesis. These findings may provide a foundation for the development of novel hypoglycemic medications derived from flavonoids. Khorasanian et al. contribute a systematic review and meta-analysis on the effects of hesperidin supplementation on cardiovascular risk factors. Hesperidin, a flavanone, has been found to significantly reduce serum triglyceride, total cholesterol, low-density cholesterol, tumor necrosis factor-α, and systolic blood pressure in human body. However, to achieve effective reductions in insulin levels, a dose of 1,000 mg per day and a duration of at least 8 weeks are required.

Park et al. have unraveled the chemical profile of gamma-irradiated wheat hulls through untargeted metabolite analysis. They identified a total of 55 compounds, among which seven flavonolignans emerged as characteristic molecules. Further bioactivity assays revealed the anti-inflammatory effect of wheat hulls in lipopolysaccharide-treated RAW 264.7 cells, with flavonolignan showing robust potential. These findings underscore the value of wheat hulls as a source of beneficial compounds and emphasize their potential as natural health-promoting ingredients in food supplements. Zhu et al. explore the beneficial effects of blueberry anthocyanins on obesity and diabetes mellitus. The active anthocyanins identified are petunidin-3-O-galactoside, petunidin-3-O-glucoside, delphinidin-3-O-galactoside, delphinidin-3-O-glucoside, and malvidin-3-O-galactoside. These compounds demonstrate remarkable antioxidant, hypoglycemic, and hypolipidemic potentials *in vitro*. Wang et al. investigate the effect of silibinin on serum lipids, bile acids, and gut microbiota by using a methionine-choline-deficient diet-induced mice model. Silibinin, a flavonoid lignin derived from the fruits and seeds of *Silybum marianum*, has been shown to regulate gut microbiota composition. Subsequently, it decreases the serum levels of lipids and bile acids in mice through gut-liver axis. This work highlights the potential impact of silibinin on non-alcoholic steatohepatitis.

The editors hope that this Research Topic of manuscripts will further inspire interest in exploring the effects and action mechanisms of flavonoids on chronic metabolic diseases.

## Author contributions

QZ: Conceptualization, Formal analysis, Writing – original draft. YN: Conceptualization, Writing – review & editing. PL: Resources, Writing – review & editing. YZ: Supervision, Writing – review & editing.

